# Supplementary material for: Splitting sleep between the night and a daytime nap reduces homeostatic sleep pressure and enhances long-term memory
Source: Sci Rep. 2021 Mar 5;11:5275. doi: 10.1038/s41598-021-84625-8 (PMC7935993; doi:10.1038/s41598-021-84625-8)
Supplement: Supplementary file 1 — Supplementary Information. [file 41598_2021_84625_MOESM1_ESM.docx]

**Splitting sleep between the night and a daytime nap reduces homeostatic sleep pressure and enhances long-term memory**

James N. Cousins^†a,b,c^, Ruth L. F. Leong^†a^, S. Azrin Jamaluddin^a^, Alyssa S. C. Ng^a^, Ju Lynn Ong^a^, Michael W.L. Chee*^a,b^

^a^Centre for Sleep and Cognition, Yong Loo Lin School of Medicine, National University of Singapore, Singapore

^b^Neuroscience and Behavioral Disorders Program, Duke-NUS Medical School, Singapore

^c^Donders Institute for Brain, Cognition & Behaviour, Radboud University Medical Centre, 6525 EN, Nijmegen, The Netherlands

^†^Both first authors contributed equally to this work

*Corresponding author:

Dr. Michael W.L. Chee

Centre for Sleep and Cognition

Human Potential Program

Yong Loo Lin School of Medicine,

National University of Singapore

MD1, 12 Science Drive 2

Singapore 117549

Phone: (+65) 66013199

E-mail: [michael.chee@nus.edu.sg](mailto:michael.chee@nus.edu.sg)

**Materials and correspondence** requests should be directed to corresponding author M.W.L.C.

**Supplementary Materials**

***
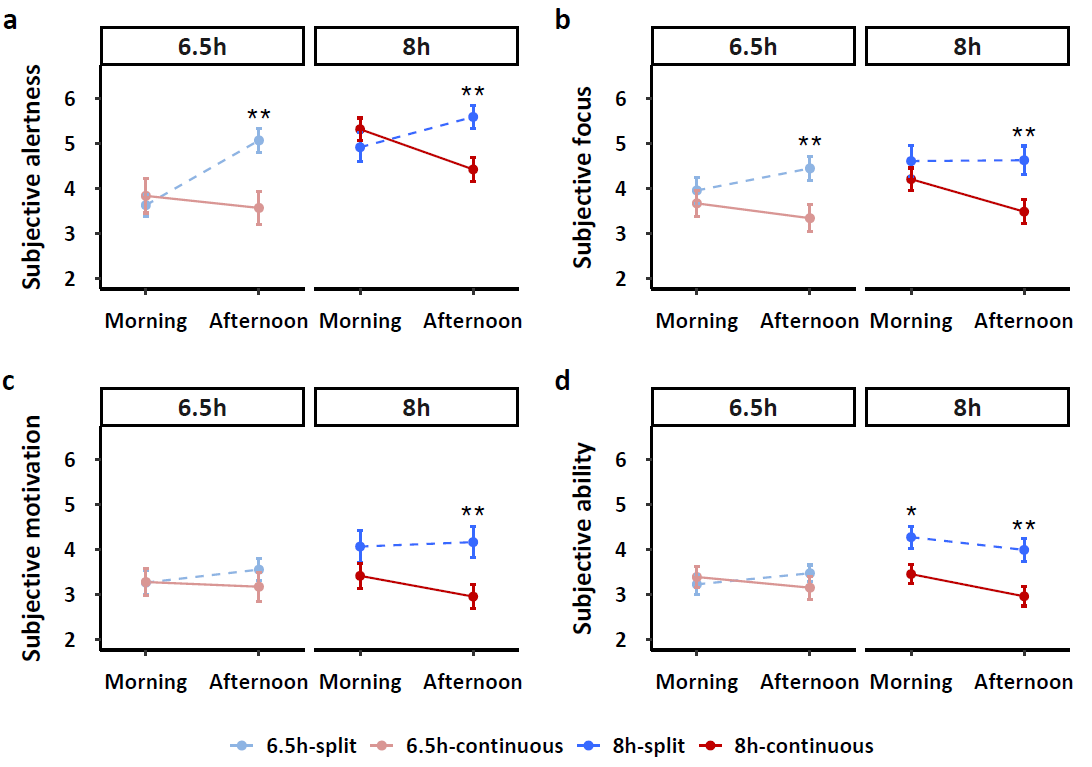
***

**Figure S1. Subjective measures during factual knowledge encoding.** Participants indicated their subjective alertness, focus, motivation, and learning ability during every learning session. **a** Subjective alertness was significantly higher in the afternoon under the split sleep schedule (6.5h: t(56)=-3.287, p=0.002; 8h: t(51)=-3.137, p=0.003) but similar across groups in the morning session. **b** A similar pattern emerged for subjective focus, which was higher for the split sleep groups in the afternoon (6.5h: t(56)=-2.754, p=0.008; 8h: t(51)=-2.782, p=0.008) but did not differ in the morning. **c** Subjective motivation did not differ at any point for the 6.5h groups, while it was significantly higher in the afternoon for the split sleep group under the 8h duration (t(51)=-2.824, p=0.007). **d** Lastly, subjective ability was similar for the 6.5h groups, while in contrast the 8h groups differed significantly in the morning (t(51)=-2.580, p=0.013) and afternoon (t(51)=-3.143, p=0.003). In summary, a split sleep schedule increased subjective alertness and focus in the afternoon for both the 6.5h and 8h durations, but only increased subjective motivation and learning ability in the afternoon for the 8h groups. Mean ± standard error of the mean (SEM). ***p < 0.001, **p < 0.01, *p < 0.05.

**Table S1.** Main effects and interactions for a mixed ANOVA including the factors of schedule (split/continuous), duration (6.5h/8h), and time (morning/afternoon) for participants’ subjective impressions of their alertness, focus, motivation, and learning ability during factual knowledge encoding.

|  | Subjective measures | | | | | | |
| --- | --- | --- | --- | --- | --- | --- | --- |
|  | **F_Schedule_** | **F_Duration_** | **F_Time_** | **F_Schedule_**  **_x Duration_** | **F_Schedule_**  **_x Time_** | **F_Duration_**  **_x Time_** | **F_Schedule_**  **_x Duration_**  **_x Time_** |
| Alertness | 2.829 | 13.790*** | 3.956* | 0.221 | 57.934*** | 9.875** | 0.100 |
| Focus | 6.664* | 1.853 | 2.869 | 0.019 | 22.729*** | 6.513* | 0.053 |
| Motivation | 3.383 | 1.216 | 0.383 | 1.710 | 8.610** | 2.823 | 0.249 |
| Learning ability | 4.680* | 2.474 | 6.770* | 3.856 | 6.591* | 7.109** | 0.882 |

****p* < .001, ***p* < .01, and **p* < .05

**Table S2:** Correlations between nap parameters and memory encoding.

|  | **6h-split** | | **8h-split** | |
| --- | --- | --- | --- | --- |
|  | ***r*** | ***p*** | ***r*** | ***p*** |
| **M1_5_:**  **Picture encoding (A’)** |  |  |  |  |
| Total sleep time (min) | 0.141 | 0.474 | -0.380 | 0.100 |
| N1 (min) | -0.118 | 0.549 | 0.108 | 0.649 |
| N2 (min) | -0.025 | 0.898 | 0.218 | 0.357 |
| N3 (min) | 0.230 | 0.240 | -0.329 | 0.157 |
| Rapid eye movement sleep (min) | -0.111 | 0.575 | -0.238 | 0.313 |
| Spindle count (12-15 Hz) | -0.066 | 0.738 | 0.018 | 0.940 |
| **M2_1_ and M2_3_ (averaged):**  **Factual knowledge task - Afternoon (certain memory)** |  |  |  |  |
| Total sleep time (min) | 0.104 | 0.591 | 0.060 | 0.790 |
| N1 (min) | 0.063 | 0.747 | -0.311 | 0.159 |
| N2 (min) | 0.106 | 0.583 | -0.162 | 0.471 |
| N3 (min) | -0.216 | 0.261 | -0.122 | 0.587 |
| Rapid eye movement sleep (min) | 0.189 | 0.325 | -0.055 | 0.807 |
| Spindle count (12-15 Hz) | 0.115 | 0.560 | -0.089 | 0.686 |
| Note: Given the nap benefit to afternoon memory under split sleep schedules, we correlated performance on each memory task with sleep parameters derived from polysomnography during the naps. Each task was correlated with the nap or naps that occurred shortly before performance of the task. | | | | |

**Table S3:** Correlations between SWE in the 1^st^ h and memory encoding.

|  | **6.5h-split** | | **8h-split** | | **6.5h-continuous** | | **8h-continuous** | |
| --- | --- | --- | --- | --- | --- | --- | --- | --- |
|  | ***r*** | ***p*** | ***r*** | ***p*** | ***r*** | ***p*** | ***r*** | ***p*** |
| **R1_1_:**  Picture encoding (A’) | -0.369 | 0.064 | 0.227 | 0.336 | -0.079 | 0.713 | -0.349 | 0.121 |
| **M2_1_ and M2_3_ (averaged):**  Factual knowledge task (certain memory) | 0.137 | 0.478 | 0.126 | 0.597 | -0.150 | 0.454 | -0.036 | 0.860 |

**Table S4**. Sleep architecture (%) and spindle density in the 6.5h-split, 8h-split, 6.5-continuous and 8h-continuous groups during the baseline, manipulation, and recovery period, measured with polysomnography.

|  |  | **6.5-split** | | **8h-split** | | **6.5h-continuous** | | **8h-continuous** | |
| --- | --- | --- | --- | --- | --- | --- | --- | --- | --- |
|  |  | Mean | SD | Mean | SD | Mean | SD | Mean | SD |
| **B2** | Nocturnal |  |  |  |  |  |  |  |  |
|  | N1, % | 2.44 | 1.74 | 2.30 | 1.72 | 1.80 | 1.31 | 2.60 | 2.13 |
|  | N2, % | 52.82 | 5.21 | 52.14 | 3.96 | 51.58 | 7.49 | 50.70 | 6.54 |
|  | N3, % | 23.00 | 4.50 | 24.39 | 6.12 | 24.88 | 6.33 | 25.02 | 4.75 |
|  | REM, % | 21.77 | 3.87 | 21.18 | 3.56 | 21.74 | 3.91 | 21.67 | 4.47 |
|  | TST, min | 483.22 | 27.09 | 488.90 | 54.26 | 475.34 | 70.81 | 489.10 | 47.57 |
|  | Spindle density | 3.50 | 0.50 | 3.42 | 0.62 | 3.38 | 0.45 | 3.27 | 0.64 |
| **M1_1_** | Nocturnal |  |  |  |  |  |  |  |  |
|  | N1, % | 1.36 | 1.20 | 2.20 | 2.32 | 1.49 | 1.02 | 2.10 | 1.35 |
|  | N2, % | 45.90 | 7.29 | 48.53 | 5.76 | 48.87 | 8.97 | 51.41 | 4.68 |
|  | N3, % | 35.04^c^ | 6.60 | 31.87^d^ | 6.00 | 30.96^c^ | 9.00 | 27.80^d^ | 4.66 |
|  | REM, % | 17.70 | 6.23 | 17.41 | 2.90 | 18.68 | 4.82 | 18.69 | 4.61 |
|  | TST, min | 271.33^ac^ | 16.41 | 364.08^ad^ | 19.62 | 357.14^bc^ | 14.59 | 438.09^bd^ | 27.02 |
|  | Spindle density | 3.34 | 0.60 | 3.38 | 0.59 | 3.34 | 0.53 | 3.49 | 0.54 |
|  | Nap |  |  |  |  |  |  |  |  |
|  | N1, % | 2.97 | 3.13 | 2.99 | 3.86 | - | - | - | - |
|  | N2, % | 45.55 | 16.65 | 47.26 | 13.30 | - | - | - | - |
|  | N3, % | 38.27 | 18.49 | 39.40 | 18.39 | - | - | - | - |
|  | REM, % | 13.20 | 13.78 | 10.34 | 12.49 | - | - | - | - |
|  | TST, min | 70.91 | 15.70 | 76.08 | 6.66 | - | - | - | - |
|  | Spindle density | 3.09 | 0.49 | 3.24 | 0.36 | - | - | - | - |
| **M1_3_** | Nocturnal |  |  |  |  |  |  |  |  |
|  | N1, % | 1.75 | 2.76 | 1.70 | 1.46 | 1.00 | 0.90 | 1.66 | 1.47 |
|  | N2, % | 47.24^a^ | 7.19 | 51.63^a^ | 7.33 | 46.50^b^ | 7.57 | 51.91^b^ | 6.90 |
|  | N3, % | 32.05^a^ | 6.51 | 26.67^a^ | 9.13 | 31.71^b^ | 6.29 | 26.06^b^ | 5.42 |
|  | REM, % | 18.96 | 5.21 | 20.00 | 4.47 | 20.78 | 5.36 | 20.38 | 4.55 |
|  | TST, min | 265.69^ac^ | 17.10 | 348.52^ad^ | 18.36 | 366.61^bc^ | 9.54 | 441.34^bd^ | 27.48 |
|  | Spindle density | 3.51 | 0.49 | 3.59 | 0.43 | 3.32 | 0.48 | 3.67 | 0.47 |
|  | Nap |  |  |  |  |  |  |  |  |
|  | N1, % | 2.50 | 1.75 | 3.09 | 3.37 | - | - | - | - |
|  | N2, % | 45.97 | 14.76 | 45.56 | 15.39 | - | - | - | - |
|  | N3, % | 39.23 | 12.41 | 40.52 | 16.56 | - | - | - | - |
|  | REM, % | 12.31 | 10.50 | 10.83 | 9.52 | - | - | - | - |
|  | TST, min | 79.45 | 3.92 | 76.54 | 12.92 | - | - | - | - |
|  | Spindle density | 3.06 | 0.52 | 2.94 | 0.99 | - | - | - | - |
| **M1_5_** | Nocturnal |  |  |  |  |  |  |  |  |
|  | N1, % | 1.08 | 1.33 | 1.50 | 1.13 | 1.10 | 1.27 | 1.42 | 0.82 |
|  | N2, % | 46.85^a^ | 7.09 | 51.82^a^ | 5.84 | 46.84^b^ | 8.54 | 52.18^b^ | 5.09 |
|  | N3, % | 32.35^a^ | 7.59 | 26.87^a^ | 6.41 | 32.52^b^ | 7.85 | 25.45^b^ | 4.81 |
|  | REM, % | 19.72 | 6.25 | 19.82 | 5.49 | 19.53 | 5.42 | 20.95 | 4.13 |
|  | TST, min | 271.50^ac^ | 15.47 | 354.19^ad^ | 18.76 | 365.24^bc^ | 15.17 | 449.86^bd^ | 27.20 |
|  | Spindle density | 3.52^c^ | 0.49 | 3.49 | 0.51 | 3.23^c^ | 0.48 | 57 | 0.41 |
|  | Nap |  |  |  |  |  |  |  |  |
|  | N1, % | 4.33 | 5.80 | 2.09 | 4.04 | - | - | - | - |
|  | N2, % | 41.17 | 12.55 | 46.49 | 12.19 | - | - | - | - |
|  | N3, % | 36.60 | 15.61 | 41.14 | 16.08 | - | - | - | - |
|  | REM, % | 17.90^a^ | 12.54 | 10.28^a^ | 9.78 | - | - | - | - |
|  | TST, min | 79.21 | 6.24 | 76.48 | 8.42 | - | - | - | - |
|  | Spindle density | 3.09 | 0.48 | 3.24 | 0.40 | - | - | - | - |
| **R1_1_** | Nocturnal |  |  |  |  |  |  |  |  |
|  | N1, % | 1.41 | 0.68 | 2.35 | 1.54 | 1.55^b^ | 1.04 | 2.88^b^ | 4.01 |
|  | N2, % | 54.80^c^ | 7.23 | 55.64 | 5.89 | 50.00^bc^ | 5.87 | 53.06^b^ | 5.59 |
|  | N3, % | 32.05^c^ | 6.51 | 20.28 | 6.40 | 25.03^c^ | 5.31 | 23.44 | 5.46 |
|  | REM, % | 25.00 | 4.82 | 21.72 | 4.32 | 23.43 | 4.90 | 20.63 | 5.90 |
|  | TST, min | 492.52 | 34.29 | 460.04 | 102.49 | 499.63 | 71.44 | 480.10 | 69.97 |
|  | Spindle density | 3.62 | 0.38 | 3.48 | 0.53 | 3.44 | 0.53 | 3.60 | 0.43 |
| **M2_1_** | Nocturnal |  |  |  |  |  |  |  |  |
|  | N1, % | 1.06 | 0.94 | 1.86 | 1.34 | 1.44^b^ | 1.54 | 2.16^b^ | 1.59 |
|  | N2, % | 47.08 | 5.19 | 50.10 | 5.58 | 48.03^b^ | 9.19 | 51.89^b^ | 5.54 |
|  | N3, % | 33.38 | 5.67 | 29.62^d^ | 6.17 | 30.26^b^ | 7.71 | 24.86^bd^ | 4.26 |
|  | REM, % | 18.47 | 4.73 | 18.43 | 5.77 | 20.21 | 4.01 | 21.09 | 4.14 |
|  | TST, min | 276.52^ac^ | 9.65 | 358.13^ad^ | 14.24 | 362.52^bc^ | 13.60 | 440.83^bd^ | 26.14 |
|  | Spindle density | 3.38 | 0.46 | 3.50 | 0.52 | 3.32 | 0.51 | 3.54 | 0.35 |
|  | Nap |  |  |  |  |  |  |  |  |
|  | N1, % | 3.67 | 5.02 | 1.80 | 2.60 | - | - | - | - |
|  | N2, % | 42.18 | 12.55 | 43.34 | 11.98 | - | - | - | - |
|  | N3, % | 41.68 | 17.77 | 39.70 | 16.20 | - | - | - | - |
|  | REM, % | 12.47 | 12.03 | 15.16 | 10.75 | - | - | - | - |
|  | TST, min | 78.10 | 5.33 | 75.89 | 5.73 | - | - | - | - |
|  | Spindle density | 3.06 | 0.50 | 3.30 | 0.47 | - | - | - | - |
| **M2_3_** | Nocturnal |  |  |  |  |  |  |  |  |
|  | N1, % | 1.20 | 1.35 | 1.68 | 1.85 | 1.05^b^ | 0.98 | 2.10^b^ | 1.64 |
|  | N2, % | 48.47^a^ | 8.43 | 54.02^a^ | 5.58 | 46.49^b^ | 6.88 | 51.21^b^ | 5.64 |
|  | N3, % | 30.45^a^ | 6.93 | 25.47^a^ | 6.30 | 31.85^b^ | 6.62 | 25.98^b^ | 4.51 |
|  | REM, % | 19.95 | 6.19 | 18.84 | 5.11 | 20.61 | 4.70 | 20.71 | 3.14 |
|  | TST, min | 275.38^ac^ | 10.50 | 351.10^ad^ | 20.00 | 370.57^bc^ | 9.31 | 446.75^bd^ | 26.59 |
|  | Spindle density | 3.46 | 0.41 | 3.57 | 0.50 | 3.30 | 0.50 | 3.61 | 0.42 |
|  | Nap |  |  |  |  |  |  |  |  |
|  | N1, % | 2.61 | 2.63 | 10.18 | 38.74 | - | - | - | - |
|  | N2, % | 41.81 | 11.45 | 41.81 | 11.45 | - | - | - | - |
|  | N3, % | 39.29 | 12.51 | 36.47 | 18.92 | - | - | - | - |
|  | REM, % | 16.29 | 13.65 | 27.68 | 47.65 | - | - | - | - |
|  | TST, min | 79.45 | 5.74 | 72.52 | 17.45 | - | - | - | - |
|  | Spindle density | 3.02 | 0.47 | 3.29 | 0.58 | - | - | - | - |
| **R2_1_** | Nocturnal |  |  |  |  |  |  |  |  |
|  | N1, % | 1.57 | 1.28 | 2.17 | 1.77 | 1.23 | 0.66 | 1.80 | 1.23 |
|  | N2, % | 56.24^c^ | 6.70 | 54.35 | 5.89 | 50.77^bc^ | 6.98 | 54.19^b^ | 4.34 |
|  | N3, % | 18.23^c^ | 4.69 | 19.76 | 6.29 | 23.04^c^ | 6.55 | 22.81 | 3.62 |
|  | REM, % | 23.95 | 6.47 | 23.72 | 3.38 | 24.96 | 5.21 | 21.20 | 4.46 |
|  | TST, min | 484.55 | 30.41 | 480.27 | 30.41 | 500.46 | 27.92 | 494.14 | 26.17 |
|  | Spindle density | 3.64^c^ | 0.39 | 3.67 | 0.37 | 3.37^c^ | 0.48 | 3.61 | 0.43 |

Note. SD = standard deviation; N1 = stage 1 sleep; N2 = stage 2 sleep; SWS = slow-wave sleep; REM = rapid-eye movement sleep; TST = total sleep time. Spindle density, spindles/min.

^a^Significant difference between the 6.5-split and 8h-split sleep groups (independent-samples t-test, *p* < .05)

^b^Significant difference between the 6.5h-continuous and 8h-continuous sleep groups (independent-samples t-test, *p* < .05)

^c^Significant difference between the 6.5h-split and 6.5h-continuous sleep groups (independent-samples t-test, *p* < .05)

^d^Significant difference between the 8h-split and 8h-continuous sleep groups (independent-samples t-test, *p* < .05)

**Picture Encoding performance on M1_5_**

Participants made judgments regarding whether pictures contained a building or not during the encoding session. This ensured that encoding was incidental and attention was maintained throughout. Performance was close to ceiling for this task (94-98% correct), while any trials that were incorrect were removed from subsequent analyses of memory. Group differences on this measure may indicate impaired focus and attention, but the task is very easy and therefore we did not predict a great deal of variation across groups. Moreover, the high level of performance and small range between groups makes it difficult to infer whether any differences were meaningful. A 2x2 ANOVA with sleep schedule (split/continuous) and duration (6.5h/8h) showed a significant main effect of schedule (F(1,107)=7.663, p=0.007). Splitting sleep led to significantly higher accuracy under both 8h (8h-continuous vs. 8h-split: t(51)=2.654, p=0.011, Cohen’s d (*d*)=0.732) and 6.5h sleep durations (6.5h-continuous vs. 6.5h-split: t(56)=2.230, p=0.030, *d*=0.586). There was no significant main effect of duration (F(1,107)=1.322, p=0.253) and no schedule*duration interaction (F(1,107)=0.468, p=0.495). These findings may reflect relatively impaired attention under the continuous sleep schedules.

**Equation S1:** Calculation for A’ where *H* indicates the hit rate and *F* indicates the false alarm rate.

|  | $A^{'}=\left\{ \begin{aligned} .5+\frac{(H-F)(1+H-F)}{4H(1-F)} \mathrm{when} H F \\ .5-\frac{\left( F-H \right)\left( 1+F-H \right)}{4F\left( 1-H \right)} \mathrm{when} H F \end{aligned} \right.$ | (1) |
| --- | --- | --- |
